# Mothers’ Perceived Neighbourhood Environment and Outdoor Play of 2- to 3.5-Year-Old Children: Findings from the Healthy Beginnings Trial

**DOI:** 10.3390/ijerph14091082

**Published:** 2017-09-18

**Authors:** Huilan Xu, Li Ming Wen, Louise L. Hardy, Chris Rissel

**Affiliations:** 1 Sydney School of Public Health, University of Sydney, Sydney, NSW 2006, Australia; LiMing.Wen@sswahs.nsw.gov.au; 2 Health Promotion Unit, Sydney Local Health District, Sydney, NSW 2050, Australia; 3 School of Public Health, Fudan University, Shanghai 200433, China; 4 Prevention Research Collaboration, Sydney School of Public Health, University of Sydney, Sydney, NSW 2006, Australia; Louise.hardy@sydney.edu.au (L.L.H.); Chris.rissel@sydney.edu.au (C.R.)

**Keywords:** neighbourhood environment, outdoor play, physical activity, young children, perceptions

## Abstract

Background: This study aims to investigate whether mothers’ perceived neighbourhood environment is associated with outdoor playtime of 2- to 3.5-year-old children. Methods: Cross-sectional analyses were conducted using data from the Healthy Beginnings Trial (HBT). Data on children’s outdoor playtime and mothers’ perceived neighbourhood environment were collected through face-to-face interviews with mothers when their children were 2 and 3.5 years old. Walk score was obtained from a publicly available website and population density data were obtained from Australian Census data. Multiple logistic regression models were built to investigate these associations. Results: A total of 497 and 415 mother-child dyads were retained at 2 years and 3.5 years. After adjusting for intervention group allocation and other confounding factors, at 2 years, mothers’ perceptions that ‘the neighbourhood is a good place to bring up children’, ‘it is safe to play outside during the day’, and ‘there are good parks or playgrounds in neighbourhood’ were positively associated with children’s outdoor playtime. At 3.5 years, living in a free-standing house was associated with more children’s outdoor playtime. Conclusions: Children may benefit from living in a neighbourhood that supports active lifestyle. Improving social and physical environments in neighbourhoods could be an important strategy for improving young children’s physical activity.

## 1. Introduction

The high prevalence of overweight and obesity in children under the age of five years is a global public health issue [1,2,3,4]. Insufficient physical activity contributes to childhood overweight and obesity [5,6,7,8]. The current Australian physical activity guidelines recommend 1- to 5-year-old children should be physically active for at least three hours per day [9]. The Australian Health Survey 2011–2012 showed that 28% of children aged between 2 to 4 years did not meet these guidelines [10]. Thus, promoting physical activity becomes an important strategy to prevent early onset of childhood overweight and obesity. Besides weight control, regular physical activity, especially outdoor play, can also help to promote children’s physical health as well as self-regulation, cognitive, and socio-emotional capacities [11,12,13]. Understanding the factors associated with young children’s physical activity can inform the development of early interventions to increase physical activity.

The socio-ecological model emphasises that individual health behaviours are influenced by different levels of environmental factors, including social-economic, physical, and policy environment [14,15]. Neighbourhoods are the primary setting where people’s daily activities take place. Hence, the features of the social or physical environment in neighbourhoods where families live may influence people’s physical activity. Substantial evidence shows that the neighbourhood environment plays an important role in physical activity engagement in children and adults [16,17,18]. Social disorder and the built environment in neighbourhood have been the main focus of previous studies. The most commonly examined features of the environment include safety (social disorder aspect), availability or accessibility of recreation facilities, road safety, walkability, residential density, and mixed land use. However, little is known regarding the influence of neighbourhood environmental characteristics on the physical activity of children under 5 years of age.

The nature of physical activity in young children is different from that in older school-age children or adults. While walking, sports and structured physical activities are common physical activities for older children and adults, free play or outdoor play is the primary form of physical activity for young children [19]. For children between the ages of 2 to 3.5 years, outdoor play is usually free play without goals set by adults, but under parental or carer’s supervision [20]. It can be expected that the neighbourhood environment influences the physical activity of young children in different ways. In contrast to older children who have more autonomy to arrange their daily activities such as physical activity in leisure time, young children are more dependent on their parents for such activities. It is possible that parents’ perceptions of their neighbourhood environment have greater influences on young children’s physical activities than the actual environment. Therefore, Davison and Lawson suggested that research should include both subjectively perceived and objectively measured characteristics of the environment [16]. In addition, the type of accommodation and the number of vehicles in a household, which to some extent, reflect a family’s socio-economic circumstances, are also expected to have an impact on young children’s physical activity [21]. Yet, studies regarding the associations of neighbourhood environments with the physical activity of young children are sparse and the results are inconsistent [22,23,24,25,26,27]. For example, while some studies found no association between neighbourhood environmental factors, such as walkability, road safety, and yard space, with children’s physical activity [23,26], other studies have found traffic safety, public housing, and neighbourhood physical disorder were associated with children’s outdoor play [24,25]. Hence, there is a need to further investigate the associations between the neighbourhood environment and young children’s physical activity.

Outdoor play is strongly related to young children’s physical activity [28,29]. It is often used as a surrogate measure of young children’s physical activity [28]. Given the potential influence of the environment on young children’s physical activity, the present study aims to investigate whether mothers’ perceived neighbourhood environmental factors, the type of accommodation, the number of vehicles in a household, walkability, and population density are associated with the outdoor playtime of 2 to 3.5-year-old children.

## 2. Materials and Methods

### 2.1. Study Design

Cross-sectional analyses were conducted using data extracted from the Healthy Beginnings Trial (HBT). A detailed research protocol and the main findings have been published elsewhere [30,31,32]. Briefly, HBT was a 5-year randomized controlled trial that assessed the effectiveness of a staged home-based early intervention in reducing early childhood obesity over the period of 2007–2013. Pregnant mothers were recruited at 24–34 weeks of pregnancy and randomly allocated to intervention or control. The interventions started from late pregnancy (gestation age of 30–36 weeks) to children were 2 years of age. The next three years, from 2 to 5 years of age, was a follow-up phase with no intervention. The study was approved by the Ethics Review Committee of Sydney South West Area Health Service (Royal Prince Alfred Hospital Zone, X10-0312 & HREC/10/RPAH/546).

### 2.2. Study Participants

A total of 667 first-time mothers at 24–34 weeks of pregnancy were recruited from antenatal clinics at Liverpool and Campbelltown Hospitals, located in South-western Sydney, Australia. Based on Social Economic Indexes for Areas (SEIFA) rankings, the Index of Relative Socio-economic Disadvantage of this area is 1–2, indicating that it has relatively high levels of socio-economic disadvantage [33]. For this particular study, data from 497 and 415 mother-child dyads that retained at ages 2 and 3.5 years were analysed.

### 2.3. Data Collection and Measures

All data were collected by face-to-face interviews with each mother at their home by trained research nurses at baseline (30 to 36 weeks of pregnancy), and at 2, and 3.5 years follow-up. For the present study, the outcomes were children’s outdoor playtime at 2 and 3.5 years; the main study factors were neighbourhood environment factors, including mothers’ perception of the neighbourhood environment, and the type of accommodation (i.e., apartment, house), the number of vehicles in household, walkability, and population density at 2 and 3.5 years, respectively. Mothers’ demographics were collected at baseline, 2 and 3.5 years respectively.

#### 2.3.1. Children’s Outdoor Playtime

Children’s outdoor playtime was assessed using two valid survey questions from Burdette et al.’s study [28]. The mother was asked how much time her child spent playing outdoors on a typical weekday and on a typical weekend day. Outdoor playtime was then dichotomised as ‘<2 h/day’ and ‘≥2 h/day’ (based on the median outdoor playtime 2 h/day during weekdays. The current Australian physical activity guidelines recommend 1- to 5-year-old children should be physically active for at least three hours per day, which includes outdoor and indoor physical activities). Full-time employed mothers were asked to provide their estimates of child’s outdoor playtime based on the time when they were with the child.

#### 2.3.2. Neighbourhood Environment 

Mothers’ perception of their neighbourhoods was assessed by using the questions from Growing up in Australia: The Longitudinal Study of Australian Children [34]. All questions used in that study were pre-tested. The mother was asked about her general perception of the neighbourhood being a good place to bring up children. The response options were ‘very good’, ‘good’, ‘fair’, ‘poor’, and ‘very poor’. The response was dichotomised as ‘yes’ and ‘no’, with ‘yes’ referring to ‘good’ and ‘very good’, and ‘no’ referring to from ‘fair’ to ‘very poor’. For mothers’ perceived safety of their neighbourhoods, the mother was asked how strongly does she agree or disagree with four statements about her neighbourhood. They were: (1) ‘This is a safe neighbourhood’; (2) ‘There are good parks, playgrounds and play spaces in this neighbourhood’; (3) ‘It is safe for children to play outside during the day’; and (4) ‘There is heavy traffic on my street or road’. The response options were given on a 4-point scale, ranging from ‘strongly agree’ to ‘strongly disagree’. The responses were dichotomised as ‘yes’ or ‘no’, with ‘yes’ referring to ‘safe neighbourhood’, ‘having good parks, playgrounds and play spaces in neighbourhood’, or ‘safe outdoor play’. For traffic condition, the response was dichotomised as ‘no heavy traffic’ vs. ‘heavy traffic’.

An index of neighbourhood walkability was determined from a publicly available website called ‘walkscore.com’ (www.walkscore.com) [35]. The website uses the distance of local amenities such as grocery stores, restaurants, schools, parks, and public transportations to calculate a walkability score of an individual address, a suburb, or a region. Walk score has been validated and has been used in a number of public health studies [18,36,37]. Neighbourhood walkability was measured at suburb level rather than the exact individual residential address due to data privacy requirements. The suburb in which mother and child lived was updated before each home visiting for data collection. A continuous walk score ranging from 0 (lowest walkability/car dependent) to 100 (highest walkability/walker’s paradise) was obtained for each suburb in which participants lived. A five-scale categorisation is used: Walk Score 90–100 (walker’s paradise), 70–89 (very walkable), 50–69 (somewhat walkable), 25–49 (car-dependent), and 0–24 (car-dependent) by the developers of Walk Score [35]. The walk score was further dichotomised as ‘≥50’ referring to ‘walkable neighbourhood’ and ‘≤49’ referring to ‘car-dependent neighbourhood’. Suburb level population density was obtained from Australian Census data in 2011 [38]. It was reported as the number of persons per hectare. It was further dichotomised as ‘less than mean population density’ (<18 persons/hectare) or ‘greater than mean population density’ (≥18 persons/hectare) based on the mean population density from this sample.

#### 2.3.3. The Type of Accommodation and the Number of Vehicles in a Household

The type of accommodation was assessed using question from the NSW Adult Population Health Survey [39]. The mother was asked what type of accommodation she lived in. The responses were dichotomised as ‘free-standing house’, and ‘other’ including semi-detached, terrace, unit, flat, and apartment. The number of vehicles in the household was assessed by asking mother ‘Are there any registered vehicles, whether private or company owned, used by your household and usually parked at your home overnight?’ The response was dichotomised as ‘≤1 car’ and ‘≥2 cars’ as almost half of the household had 1 car and the other half had 2 or more cars.

#### 2.3.4. Mothers’ Demographics

Questions from the NSW Child Health Survey 2001 were used to collect mothers’ demographic and socio-economic information [40]. Details of the categorisation of demographics have been published elsewhere [41].

### 2.4. Statistical Analyses

Cross-sectional analyses were conducted when children were aged 2 and 3.5 years using statistical software Stata version 13 (StataCorp LP, College Station, TX, USA) [42]. Descriptive analyses were conducted to summarise participants’ demographics. The outcome, children’s outdoor playtime, was a binary variable. Logistic regression analyses were conducted for each study factors. First, bivariate logistic regression models were built to examine the association of each study factor or each potential confounding factor with children’s outdoor playtime. Odds ratios (ORs) with a 95% confidence interval (CI) were reported. Second, multivariable logistic regression models were built to examine the association of each study factor with children’s outdoor playtime with adjustment for potential confounding factors, such as child-care attendance and mothers’ physical activity. Adjusted odds ratios (AORs) with a 95% CI were calculated. Children’s outdoor playtime on weekdays may be different from that at weekends. For those children who attended child-care services, or those mothers who were employed full-time, mother reports of outdoor playtime may affect the estimates of outdoor playtime on weekdays. Hence, all analyses were conducted for weekdays and weekends separately.

Since the HBT was a randomised controlled intervention trial, all multiple logistic regression models were adjusted for group allocation to control the intervention effect. A backward elimination approach was used to build a multivariable logistic regression model. All potential confounding factors, including demographics with *p* < 0.25 on bivariate analysis, were entered into a multiple logistic regression model that included each study factor and allocation of intervention. The least significant terms, except for the study factor and the allocation of intervention, were progressively dropped until only those with *p* < 0.05 remained. The factors excluded from the model were checked one at a time and added if they were significant or were confounding the effect of the study factor in the model. A 10% change in the odds ratio was used as the cutoff to determine confounding factors. Since walk scores and population density were measured on the suburb level, multilevel mixed-effects logistic models were built to take the clustered nature of data into account when investigating the association of walkability or population density with children’s outdoor playtime.

## 3. Results

The characteristics of the mothers and children at ages 2 and 3.5 years are shown in Table 1. With the exception of annual household income, there were no significant differences in mothers and children’s socio-demographics at 2 and 3.5 years. Compared to the participants at 2 years, significantly, more participants with higher income retained at 3.5 years. Mothers who were lost to follow-up were typically young, unmarried, had less education, were unemployed, and had a lower household income.

The results of descriptive analyses of children’s outdoor playtime and neighbourhood environment factors are shown in Table 2. The percentage of children having 2 or more than 2 h outdoor playtime per day increased from 63% to 67%. However, this increase was not statistically significant. There were no significant changes in most neighbourhood environment factors from 2 to 3.5 years of age. However, the percentage of mothers who thought it was not safe for children to play outdoors and there was heavy traffic in the neighbourhood significantly increased from 26% to 42% and from 19% to 26%, respectively.

The results of bivariate and multiple analyses are shown in Table 3 and Table 4. Most of the statistically significant associations from bivariate analyses remained significant after adjusting for allocation of intervention group and other confounding factors. At 2 years, mothers who perceived that their neighbourhoods is a good place to bring up children were more likely to have their children playing outdoor for ≥2 h/day with AOR 1.87 (95% CI 1.13–3.07) on weekdays and 1.91 (95% CI 1.12–3.27) at weekends; mothers who perceived that it is safe for children to play outside were more likely to have their children playing outdoor for ≥2 h/day with AOR 2.06 (95% CI 1.29–3.30) on weekdays, and 2.47 (95% CI 1.46–4.19) at weekends; mothers who perceived that there are good parks or playgrounds in the neighbourhood were more likely to have their children playing outdoors for ≥2 h/day with AOR 1.86 (95% CI 1.09–3.18) on weekdays, and AOR 1.83 (95% CI 1.03–3.25) at weekends. The association between the number of vehicles in a household and outdoor play was only statistically significant on weekdays (Table 4).

At age 3.5 years, children who lived in a free standing house were more likely to play outdoors for ≥2 h/day on both weekdays (AOR 2.03; 95% CI 1.17–3.51) and weekends (AOR 2.23; 95% CI 1.09–4.55); mothers who perceived that their neighbourhood is a good place to bring up children (AOR 2.96; 95% CI 1.42–6.17) and it is safe for children to play outside (AOR 1.94; 95% CI 1.02–3.70) were more likely to have their children playing outdoor for more than 2 h/day at weekends. Other neighbourhood environment factors, such as mothers’ perceived safe neighbourhood, traffic, suburb level walkability, and population density were not associated with children’s outdoor playtime at 2 and 3.5 years (Table 4).

Multiple logistic regression models also showed that from ages 2 to 3.5 years, children with Australian born mothers were more likely to play outdoors for more than 2 h per day during both weekdays and weekends. When compared to mothers who had a less formal education (i.e., completed primary school to School Certificate), mothers who had achieved a higher educational level (i.e., Higher School Certificate, Technical and Further Education, and university degree) were less likely to allow their children to play outdoors for more than 2 h per day on weekdays. However, mother’s own physical activity was not significantly associated with children’s outdoor playtime.

## 4. Discussion

The present study indicates that some factors in the neighbourhood environment could be associated with children’s outdoor playtime at ages 2 and 3.5 years. Mothers’ perceptions that the neighbourhood is a good place to bring up children and that it is safe to play outdoors were associated with children’s higher likelihood of playing outdoors. Mothers’ perception that there are good parks or playgrounds in neighbourhood was associated with children’s higher likelihood of playing outdoors at 2 years. Children living in free-standing house were more likely to play outdoors at 3.5 years.

Mothers’ perceptions that the neighbourhood is a good place to bring up children can be seen as a general view of their neighbourhood. It may reflect that the neighbourhood is safe (i.e., low crime rate), easy to access to recreational facilities, easy to travel, and shopping, etc. Similarly, mothers’ perceptions that it is safe for children to play outdoors can represent physical or social aspects of safety in neighbourhood, such as road safety, or high community cohesion and lower crime rate. There could be some overlap among these perceptions. Heavy traffic on streets or roads is one of indicators regarding road safety and may reflect mother’s concern about their children’s safety, especially when they play outside. An American study found that 5–10 year old children had a lower physical activity level when their parents were concerned about neighbourhood safety regarding both social-disorder and road safety [43]. Another American population study found that five year old children spent more hours playing outdoors and had more trips to a park or playground when their mother had higher perceptions of neighbourhood collective efficacy (i.e., mutual trust between neighbours who look out for one another) [24]. Findings from our study echoed this evidence.

However, in our study, mothers’ general view of neighbourhood safety and traffic situations were not associated with young children’s outdoor playtime. An Australian study also found that parental perceptions of neighbourhood safety (i.e., road safety, incivilities, and personal safety) was not associated with young children’s moderate-to-vigorous physical activity, but was associated with adolescents’ moderate-to-vigorous physical activity [44]. In our study sample, the proportion of mothers who perceived that outdoor play is safe and the traffic is not heavy were significantly less at 3.5 years than that at 2 years. This may show that mothers’ safety concerns change as children grow and become more mobile. A Netherlands study found that traffic volume and speed was not significantly related to outdoor play in 4 to 12 year old children, while other aspects of road safety, such as presence of zebra crossings, traffic lights, and roundabouts, were associated with outdoor play in children [25]. This indicated that other features of neighbourhood environment might have a stronger influence than traffic volume or speed, or possibly, mothers’ natural protection of young children weakens the influence of traffic.

With regard to recreational facilities, although using subjective measures of parks and playgrounds, our finding were in line with previous studies that used objective measures [22,27,45]. Parks and playgrounds provide one opportunity for children’s outdoor play. It can be expected that the availability or accessibility of parks and playgrounds were associated with outdoor play in young children. A New Zealand study used objective measures of, and teenagers’ perceived access to, parks or playgrounds. The authors found that teenagers’ perceived access to parks or playgrounds was positively associated with their self-reported physical activity, but not the objectively measured total physical activity [46]. The findings from our study and the New Zealand study might suggest that mothers’ or children’s perception of being closer to parks or playgrounds may have more influence on engaging children in outdoor play. A Netherlands study using objective measures of environment found that the number of formal outdoor play facilities per square kilometer was negatively related to outdoor play in young children, while the informal places such as sidewalks, parallel parking spaces, or grouped parking places was positively associated with outdoor play in young children [25]. The author concluded that this difference may be due to researchers examining the availability of parks or playground at the neighbourhood level, rather than focusing on the individual accessibility of parks or playgrounds that may be a more proximal factor in relation to children’s outdoor play. Sidewalks or parallel parking spaces are usually closer to home and can be informal play spaces for children. Their findings suggested that proximity to play places have more influence on motivating children to play outdoors.

While many studies found that a higher walkability was positively associated with adults’ transformational or recreational walking and cycling [18,47], results from studies for older children were inconsistent. Some studies found that a higher walkability was related to more physical activity, including active transportation to school in children [27,48,49,50,51], some did not find such an association [46,52], and others found a negative association. A Belgian study found that a lower walkability and longer distance to school was associated with more physical activity in adolescents [53]. Low street connectivity (i.e., more cul-de-sacs) is a characteristic of a low walkable neighbourhood. Meanwhile, it is a characteristic of lower traffic and higher road safety. Many studies found that children living in cul-de-sac neighbourhoods were more active or spent more time playing in neighbourhoods [45,54,55]. Our study found suburb level walk score was not associated with young children’s outdoor play. Partly, it might be a result of using of suburb level walkability rather than individual household walkability. The cluster nature of data significantly reduced the statistical power to detect the potential association between walkability and children’s outdoor play. On the other hand, neighbourhood walkability usually represents opportunities for walking in everyday life. It is more relevant to daily life of adults or adolescents rather than young children, as the primary form of physical activity of young children is play not walking. Therefore, the effect of neighbourhood walkability on adults may not be generalized to young children.

Population density is the population per unit of land. Intuitively, when the population density is high, the play space can be affected which in turn affect children’s outdoor play. However, our study did not support this hypothesis. Again, it is possible due to the using of suburb level population density, and the cluster nature of data reduced the statistical power to detect the effect of population density. Studies about the influence of population density on children’s physical activity or outdoor play are sparse. Inconsistent findings resulted from two American studies. One study found that a higher population density was associated with higher rates of walking and biking to school in school-age children [56], and another study did not find such an association [57].

Our study found that the type of accommodation children lived in (free standing house) was positively associated with outdoor playtime at 3.5 years of age. For children who live in a free standing house, it is possible that they are more likely to play in the front or back yard of their house. In Australia, there is an increasing urban trend towards high-rise apartment housing due to population increases. Future neighbourhood design should include sufficient green and outdoor play spaces in high housing density areas.

To some extent, the number of cars in a household may reflect the social-economic status of a family, which could influence children’s physical activity. One study found a higher family social-economic status was associated with higher physical activity levels in children younger than 5 years of age [26]. Children from households that had two or more vehicles were less likely playing outdoors for more than 2 h per day on weekdays at 2 years of age. A UK study revealed that more household cars in use were associated with lower physical activity level in seven year old children [21]. It is possible that having two or more vehicles in a household indicates both parents are working, which could affect outdoor playtime of their children. Further research is needed to explore this phenomenon.

The differences in findings between 2 and 3.5 years of age, and between weekdays and weekend days, indicate that different features or aspects of neighbourhood environments are associated with outdoor play of children at different ages and times of the week. As discussed above, mothers’ concerns and perceptions of neighbourhood environment can change along with children’s growth, which may also contribute to the differences.

The findings of the present study offered some insight into the associations of the neighbourhood environment with outdoor playtime of young children. Neighbourhood design should consider safety, and good parks or playgrounds for young children’s outdoor play. However, they should be viewed in the light of some limitations. For example, a loss to follow-up may cause selection bias. The cross-sectional analysis precludes the attribution of causality. Recall bias might be caused by the nature of mothers’ reporting their child’s outdoor playtime, especially for those children who attended child-care services or those mothers who were employed full-time, such self-reports may affect the estimates of outdoor playtime on weekdays. Although questions regarding neighbourhood environments were extracted from Growing up in Australia: The Longitudinal Study of Australian Children and were pre-tested, no reliability or validity scores are available. All environment variables were dichotomized due to non-normal distributions, which may have caused some information to be lost. In addition, some neighbourhood or home physical environment factors were not included in this study, such as weather or seasons, crime, or yard size. It is also worth noting that the study was conducted in South-western Sydney, Australia, an area with a relatively low socio-economic level, which could limit the generalizability of the study.

## 5. Conclusions

This study found that apart from socio-economic factors, some mothers’ perceived neighbourhood environment (both social and physical) factors were also associated with outdoor playtime of children at ages 2 and 3.5 years. It supports the socio-ecological model that physical activity behaviour in young children is influenced by different levels and aspects of environment that surround them. Children may benefit from living in a safer and healthier neighbourhood that supports active lifestyle. Improving the social and physical environment in neighbourhoods could be an important strategy for improving the physical activity of young children.

## Figures and Tables

**Table 1:** Characteristics of participating mothers and children at ages 2 and 3.5 years.

Variables	Child’s Age	
	2 Years Old	3.5 Years Old
	*n* (%)	*n* (%)
**Mother’s country of birth**		
Other	175 (35)	151 (36)
Australia	321 (65)	263 (64)
**Language spoken at home**		
English	446 (90)	376 (91)
Other	48 (10)	36 (9)
**Mother’s age at late pregnancy**		
≤24	185 (37)	140 (34)
25–29	176 (36)	153 (37)
≥30	136 (27)	122 (29)
**Mother’s education**		
Completed primary school to School Certificate Certificate	82 (17)	57 (14)
HSC to TAFE certificate or diploma *	280 (57)	234 (56)
University	133 (27)	123 (30)
**Mother’s marital status**		
Never married	65 (13)	59 (14)
Married or de-facto partner	428 (87)	354 (86)
**Mother’s employment status**		
Unemployed/other	235 (48)	179 (43)
Employed	258 (52)	236 (57)
**Annual household income (AUD)**		
<$40,000	79 (18)	50 (14)
$40,000–79,999	176 (42)	117 (31)
≥$80,000	165 (39)	206 (55)
**Child sex**		
Male	248 (50)	213 (51)
Female	249 (50)	202 (49)

Note: sample size is not necessarily 497 and 415 at ages 2 and 3.5 years due to missing values. * HSC = Higher School Certificate (year 12), TAFE = Technical and Further Education.

**Table 2:** Descriptive statistics of study factors and outcomes at ages 2 and 3.5 years.

Study Outcome and Factors	2 Years *n* (%)	3.5 Years *n* (%)	*p*
**Child outdoor playtime**			0.126
<2 h/day	181 (37)	135 (33)	
≥2 h/day	305 (63)	379 (67)	
**Good neighbourhood to bring up child**			0.418
No	99 (20)	74 (18)	
Yes	395 (80)	339 (82)	
**Safe neighbourhood**			0.875
No	48 (10)	39 (9)	
Yes	443 (90)	373 (91)	
**Safe outdoor play**			<0.0001
No	125 (26)	174 (42)	
Yes	358 (74)	236 (58)	
**Good parks or playgrounds**			0.939
No	83 (17)	68 (17)	
Yes	413 (83)	343 (83)	
**Traffic in neighbourhood**			0.014
Heavy traffic	95 (19)	108 (26)	
Not heavy traffic	398 (81)	306 (74)	
**Walk score**			0.563
≤49 (car-dependent)	237 (55)	197 (57)	
>50 (walkable)	195 (45)	149 (43)	
**Population density**			0.135
<18/hatch (mean)	232 (50)	204 (55)	
≥18/hatch (mean)	234 (50)	167 (45)	
**Accommodation type**			0.083
Unit/townhouse	118 (24)	79 (19)	
Free standing house	377 (76)	335 (81)	
**Number of vehicles in use**			0.0002
0 to 1 car	228 (47)	142 (35)	
≥2 cars	260 (53)	269 (65)	

**Table 3:** Bivariate logistic regression analysis of the associations of neighbourhood environment factors with outdoor playtime of 2- to 3.5-year-olds.

Variables	Child Outdoor Playtime ≥2 h/day			
	At Age 2 Years		At Age 3.5 Years	
	Weekday OR (95% CI)	Weekend Day OR (95% CI)	Weekday OR (95% CI)	Weekend Day OR (95% CI)
**Good neighbourhood to bring up child**				
No (ref)	1.0	1.0	1.0	1.0
Yes	1.66 (1.06–2.60)	1.66 (1.00–2.76)	1.17 (0.69–1.98)	2.08 (1.09–3.96)
**Safe neighbourhood**				
No (ref)	1.0	1.0	1.0	1.0
Yes	1.42 (0.78–2.58)	1.81 (0.94–3.49)	0.94 (0.46–1.92)	0.94 (0.35–2.51)
**Safe outdoor play**				
No (ref)	1.0	1.0	1.0	1.0
Yes	2.21 (1.45–3.37)	2.68 (1.68–4.28)	1.47 (0.96–2.24)	1.84 (1.04–3.25)
**Traffic in neighbourhood**				
Heavy traffic (ref)	1.0	1.0	1.0	1.0
No heavy traffic	1.41 (0.89–2.23)	1.70 (1.01–2.84)	0.81 (0.52–1.31)	1.41 (0.77–2.60)
**Good parks or playgrounds**				
No (ref)	1.0	1.0	1.0	1.0
Yes	1.63 (1.01–2.64)	1.62 (0.94–2.78)	0.90 (0.51–1.59)	1.44 (0.71–2.89)
**Walk score**				
≤49 (car-dependent) (ref)	1.0	1.0	1.0	1.0
>50 (walkable)	1.07 (0.68–1.70)	0.87 (0.55–1.38)	0.83 (0.50–1.36)	1.27 (0.69–2.36)
**Population density**				
<18/hatch (mean) (ref)	1.0	1.0	1.0	1.0
≥18/hatch (mean)	0.66 (0.42–1.06)	0.78 (0.50–1.23)	1.01 (0.63–1.62)	0.88 (0.46–1.68)
**Accommodation type**				
Unit/townhouse (ref)	1.0	1.0	1.0	1.0
Free standing house	0.84 (0.61–1.45)	1.17 (0.71–1.93)	1.94 (1.17–3.21)	2.78 (1.50–5.13)
**Number of vehicles in use**				
0 to 1 car (ref)	1.0	1.0	1.0	1.0
≥2 cars	0.60 (0.41–0.87)	0.89 (0.57–1.39)	1.17 (0.76–1.81)	1.38 (0.77–2.45)

**Table 4:** Multiple logistic regression analyses of the associations of neighbourhood environment factors with outdoor playtime of 2- to 3.5-year-olds.

Variables	Child Outdoor Playtime ≥2 h/day			
	At Age 2 Years		At Age 3.5 Years	
	Weekday AOR (95% CI)	Weekend Day AOR (95% CI)	Weekday AOR (95% CI)	Weekend Day AOR (95% CI)
**Good neighbourhood to bring up child**				
No (ref)	1.0	1.0	1.0	1.0
Yes	1.87 (1.13–3.07)	1.91 (1.12–3.27)	1.30 (0.74–2.30)	2.96 (1.42–6.17)
**Safe neighbourhood**				
No (ref)	1.0	1.0	1.0	1.0
Yes	1.43 (0.72–2.81)	1.50 (0.69–3.25)	0.98 (0.46–2.09)	0.90 (0.29–2.77)
**Safe outdoor play**				
No (ref)	1.0	1.0	1.0	1.0
Yes	2.06 (1.29–3.30)	2.47 (1.46–4.19)	1.29 (0.82–2.03)	1.94 (1.02–3.70)
**Traffic in neighbourhood**				
Heavy traffic (ref)	1.0	1.0	1.0	1.0
No heavy traffic	1.39 (0.84–2.31)	1.57 (0.88–2.79)	0.80 (0.48–1.35)	1.45 (0.72–2.92)
**Good parks or playgrounds**				
No (ref)	1.0	1.0	1.0	1.0
Yes	1.86 (1.09–3.18)	1.83 (1.03–3.25)	0.92 (0.49–1.72)	1.68 (0.75–3.78)
**Walk scores**				
≤49 (car-dependent) (ref)	1.0	1.0	1.0	1.0
>50 (walkable)	1.55 (0.93–2.59)	1.29 (0.78–2.15)	0.98 (0.61–1.58)	1.52 (0.78–2.93)
**Population density**				
<18/Hach (mean) (ref)	1.0	1.0	1.0	1.0
≥18/Hatch (mean)	0.69 (0.40–1.20)	0.90 (0.53–1.52)	1.10 (0.68–1.77)	1.07 (0.54–2.14)
**Accommodation type**				
Unit/townhouse (ref)	1.0	1.0	1.0	1.0
Free standing house	0.87 (0.53–1.42)	0.84 (0.47–1.51)	2.03 (1.17–3.51)	2.23 (1.09–4.55)
**Number of vehicles in households**				
0 to 1 car (ref)	1.0	1.0	1.0	1.0
≥2 cars	0.56 (0.37–0.86)	0.84 (0.50–1.39)	1.11 (0.68–1.79)	1.22 (0.62–2.40)

Note: All models were adjusted for intervention allocation, child sex, mother’s country of birth, education level, physical activity level, and childcare attendance at 2 and 3.5 years respectively.
